# Cellular interaction of a layer-by-layer based drug delivery system depending on material properties and cell types

**DOI:** 10.2147/IJN.S153701

**Published:** 2018-04-05

**Authors:** Mandy Brueckner, Steffen Jankuhn, Eva-Maria Jülke, Uta Reibetanz

**Affiliations:** 1 Institute for Medical Physics and Biophysics, Medical Faculty, University of Leipzig, Leipzig, Germany; 2 Division of Nuclear Solid State Physics, Faculty of Physics and Geosciences, University of Leipzig, Leipzig, Germany; 3 Office for Environmental Protection and Occupational Safety, University of Leipzig, Leipzig, Germany

**Keywords:** layer-by-layer, LbL, uptake, cell viability, microparticles, microcapsules, material properties

## Abstract

**Background:**

Drug delivery systems (DDS) and their interaction with cells are a controversial topic in the development of therapeutic concepts and approaches. On one hand, DDS are very useful for protected and targeted transport of defined dosages of active agents. On the other hand, their physicochemical properties such as material, size, shape, charge, or stiffness have a huge impact on cellular uptake and intracellular processing. Additionally, even identical DDS can undergo a completely diverse interaction with different cell types. However, quite often in in vitro DDS/cell interaction experiments, those aspects are not considered and DDS and cells are randomly chosen.

**Methods and results:**

Hence, our investigations provide an insight into layer-by-layer designed microcarriers with modifications of only some of the most important parameters (surface charge, stiffness, and applied microcarrier/cell ratio) and their influence on cellular uptake and viability. We also considered the interaction of these differently equipped DDS with several cell types and investigated professional phagocytes (neutrophil granulocytes; macrophages) as well as non-professional phagocytes (epithelial cells) under comparable conditions. We found that even small modifications such as layer-by-layer (LbL)-microcarriers with positive or negative surface charge, or LbL-microcarriers with solid core or as hollow capsules but equipped with the same surface properties, show significant differences in interaction and viability, and several cell types react very differently to the offered DDS.

**Conclusion:**

As a consequence, the properties of the DDS have to be carefully chosen with respect to the addressed cell type with the aim to efficiently transport a desired agent.

## Introduction

With regard to personalized and innovative medicine, therapy strategies do not only focus on a special drug. There is also a high demand for new and adaptable drug delivery systems (DDS) aiming both at the reduction of impairments and suffering of the patients and the reduction of adverse side effects. Accordingly, in the past few decades, biomedical research has focused more and more on drug delivery research, which has become an established field in pharmaceutical science exploring multiple approaches.^1^,^2^ However, besides their primary function to carry and release active agents, cellular interaction and uptake of the DDS depend on several DDS-specific parameters: as an example, material, size, stiffness, shape, surface charge and surface modification play an important role in this matter. Also, cellular characteristics have to be considered, since phagocytosing/endocytosing properties of the same DDS design differ dramatically between the cell types. Feliu et al recently published a review illustrating the high significance and strong influence of such parameters on DDS/cell interaction by examining nano- and microcarriers. As summarized, not only the variety of DDS properties but also the experimental conditions used in DDS/cell investigations make it very difficult to compare the results and conclusions of different approaches and experiments presented in a multitude of studies. As a consequence, more comprehensive research is needed to understand the interconnectivity of DDS-specific and cellular properties.^3^

We now address this matter by investigating and comparing the influence of a selected DDS with different modifications on interaction with several cell types under comparable experimental conditions, to illustrate their strong connection. For this reason, no (model) active agents were considered, but investigations concentrated solely on the properties of the DDS and their respective target cells.

As a DDS, we focus on layer-by-layer (LbL) microcarriers, whose modularity and multifunctionality allow an easily implementable modification.^4^,^5^ Based on the alternating assembly of, for example, oppositely charged bio-polyelectrolytes, a multilayer can be build up on spherical templates of different sizes ranging from nanometer to micrometer dimensions.^6^ Different active agents can be adsorbed in defined amounts with time-controlled and sustained release kinetics by integrating them into the core or into different layers of the multilayer. While nanometer-sized LbL carriers with respective functionalization can be basically used in blood circulation, larger carriers are intended to be applied in local injections into a specific tissue.^7^–^9^

Physicochemical properties such as size, shape and charge of DDS are strongly interconnected, which means changing one property may affect others.^10^ Furthermore, it has to be considered that DDS may change their properties after internalization by cells, which are supposed to affect intracellular processing in an undesired way.^3^,^11^,^12^ We, therefore, set our focus on most important modifications such as shape/stiffness and surface charge and investigated them in a concentration- and time-dependent manner. In this context, solid polyelectrolyte-coated microparticles (PEMPs) with high stiffness as well as polyelectrolyte-coated microcapsules (PEMCs) with low stiffness and high deformability were applied. These microcarrier types also exhibit different properties in intracellular accumulation, biocompatibility, filling potential and release kinetics from multilayer or core.^13^–^20^

The surface charge of the DDS is another important parameter. We applied microcarriers, PEMPs as well as PEMCs, with two modifications: with a negative and a positive surface charge characterized by the charge of the outermost polymer layer. It is generally believed that a positive charge enhances the cellular uptake, but is considered as a more toxic environment for the cells and vice versa for negatively charged carriers.^21^

Regarding the cell lines, we focused on two cell line representatives of professional phagocytes (macrophage-like cell line U937, neutrophil granulocyte-like cell line HL-60) and one representative of non-professional phagocytes (epithelial cell line HEK293T/17). All cell types are important representatives of either immune response or basic tissue and targets for medical approaches.^22^–^25^ Importantly, they all have in common nearly comparable experimental handling conditions compared to primary cells.

In our investigations, it could be clearly demonstrated that, indeed, even the narrow spectrum of our microcarrier-cell experiments regarding cellular uptake and viability show extensive differences, which becomes evident particularly under the applied comparable experimental conditions. Even similar cell types (both professional phagocytes) are not comparable with respect to cellular uptake and impairments of viability after LbL-microcarrier application. These findings support the assumption that the microcarrier design has to be neatly adapted and optimized for applications to a specific cell type before the question of integration, transport and release of active agents arises.

## Materials and methods

### Materials

Cell lines were purchased from American Type Culture Collection (ATCC, Manassas, VA, USA): HL-60 (ATCC^®^ CCL-240™), U937 (ATCC CRL-1593.2™) and HEK293T/17 (ATCC CRL-11268™). Dulbecco’s Modified Eagle’s Medium (DMEM), fetal bovine serum (FBS), penicillin/streptomycin, trypsin, phosphate-buffered saline (PBS) without (w/o) calcium, and Roswell Park Memorial Institute (RPMI) 1640 medium were obtained from Biowest (Nuaillé, France). Retinoic acid, phorbol 12-myristate 13-acetate, poly-l-arginine hydrochloride (ARG), poly (allylamine hydrochloride) (PAH), poly (sodium 4-styrenesulfonate) (PSS), Hank’s balanced salt solution (HBSS), Triton™ X-100, Tris buffer, lithium lactate, iodonitrotetrazolium chloride (INT)/phenazine methosulfate (PMS)/β-nicotinamide adenine dinucleotide (NAD) solution, PMS and valinomycin were purchased from Sigma-Aldrich (Munich, Germany). Dextran sodium sulfate (DXS) was obtained from INC Biochemicals (Irvine, USA). Amicon stirred cell 8003 was obtained from Merck Millipore (Billerica, MA, USA). Durapore^®^ polyvinylidenfluorid (PVDF) membrane was from EMD Millipore (Billerica, MA, USA). Fluka Chemie (St Gallen, Switzerland) provided EDTA. Twenty-four-well (U937), 48-well (HL-60, HEK293T/17) and 96-well plates were purchased from Greiner Bio-One (Frickenhausen, Austria). JC10 stock solution was from Enzo Life Science (Lörrach, Germany). Eight-well Lab-Tek II chamber slides were obtained from Thermo Fisher Scientific (Waltham, MA, USA). Hoechst33342 (Hoechst) was bought from Thermo Fisher Scientific. NAD was from Carl-Roth (Karlsruhe, Germany).

### Methods

#### Preparation

Preparation and LbL coating of calcium carbonate (CaCO_3_)-microparticles

CaCO_3_-microparticles with a narrow size distribution of 5±1 µm were prepared by crystallization. According to the report of Volodkin et al, equal volumes of 0.33 M CaCl_2_ and 0.33 M Na_2_CO_3_ solution were rapidly mixed for 1 min at room temperature.^26^ After 10 min of sedimentation and CaCO_3_ precipitation, the microparticles were centrifuged for 1 min at 2,000×*g* and washed five times with distilled water.

Using the LbL technique, spherical CaCO_3_-microparticles were coated in an alternating incubation procedure with oppositely charged polyelectrolytes.^4^,^5^ As the biocompatible and biodegradable polyelectrolyte system ARG, M_w_ ≥70 kDa, and DXS, M_w_ ~40 kDa, both 1 mg/mL in 0.5 M NaCl were used. PAH, M_w_ ~56 kDa, and PSS, M_w_ ~70 kDa, both 1 mg/mL in 0.5 M NaCl served as a synthetic and nonde-gradable polyelectrolyte system assembled at inner layers for specific investigations.

Additionally, fluorescent-labeled polyelectrolytes were applied. Therefore, PAH was labeled with rhodamine isothiocyanate (RITC) as previously described.^27^ For each adsorption step, CaCO_3_-microparticles were incubated in polyelectrolyte solution (ARG, DXS, PAH or PSS) for 10 min under constant shaking. To remove the unbound polyelectrolytes, CaCO_3_-microparticles were washed three times with 0.1 M NaCl. To investigate microcarrier/cell interaction, the following coating schemes were used: [PAH/PSS]_1_-[PAH^RITC^/PSS]_2_-[ARG/DXS]_3_ or [PAH/PSS]_1_-[PAH^RITC^/PSS]_2_-[ARG/DXS]_3_-ARG. For viability investigations, the coating schemes were as follows: [ARG/DXS]_4_ or [ARG/DXS]_4_-ARG.

#### Microcapsule production

After coating CaCO_3_-microparticles with eight (viability study) or 12 (interaction study) layers, the dissolution of the CaCO_3_ core was carried out using an Amicon stirred cell 8003 with a Durapore PVDF membrane (0.65 µm). CaCO_3_-microparticles, referred to as PEMPs (polyelectrolyte microparticles) hereafter, were incubated twice in 0.5 M EDTA for 20 min. To remove the core material and EDTA, the resulting PEMCs (polyelectrolyte microcapsules) were washed three times with PBS w/o calcium. An additional layer assembly with biocompatible polyelectrolytes (ARG, DXS) was performed, respectively.

#### Cell culture and differentiation

HEK293T/17 cells, a human embryonic kidney cell line, were maintained in DMEM, supplemented with 10% heat-inactivated FBS and 100 U/mL penicillin and 0.1 mg/mL streptomycin in a humidified atmosphere of 5% CO_2_ and 37°C. The suspension cell lines HL-60 and U937 were cultured in RPMI 1640 medium containing 10% FBS and 100 U/mL penicillin and 0.1 mg/mL streptomycin. To initiate differentiation of HL-60 cells into neutrophil granulocyte-like cells, RPMI 1640 medium was complemented with 40 µM retinoic acid and cells were incubated for 30 h.^28^ To differentiate the U937 cell line into macrophage-like cells, 5×10^4^ cells were incubated in 1 mL RPMI 1640 medium with 10% FBS and 10 ng/mL phorbol 12-myristate 13-acetate for 48 h.^29^ The efficient differentiation of both cell lines, HL-60 and U937, was verified by the detection of typical morphologic and functional changes of the cells as well as characteristic antibody staining (data not shown).

#### Microcarrier/cell co-incubation

Cells were cultured in 24-well (U937) or 48-well (HL-60, HEK293T/17) plates in a humidified atmosphere depending on different cell culture conditions: 1×10^5^ differentiated HL-60 cells in 0.5 mL RPMI 1640 medium, 5×10^4^ differentiated U937 cells in 1 mL RPMI 1640 medium and 1.5×10^5^ HEK293T/17 cells in 0.5 mL DMEM, each containing 2% FBS. Both LbL-microcarriers, PEMPs as well as PEMCs, were added in microcarrier:cell (m:c) ratios of 1:1, 5:1 and 10:1 to the cells for different incubation times, which varied due to the different interaction and culture characteristics. After each incubation time point, the medium was removed and the cells were washed with PBS and incubated with trypsin to detach adherent cells and to reduce unspecific microcarrier attachment on the cell surface. Subsequently, the trypsin incubation was stopped with a medium containing 10% FBS and cells were suspended in PBS for Flow Cytometry (FCM) analysis.

#### Mitochondrial membrane potential changes

Loss of mitochondrial membrane potential was used as an early marker for apoptosis. Therefore, cells were seeded as described before and microcarriers were added in m:c ratios of 1:1, 5:1 and 10:1 for three different incubation times, which were chosen according to the microcarrier’s intracellular processing. Additionally, for each cell type and incubation time, a positive control (apoptotic cells) was induced using 6 µM valinomycin. JC10 stock solution was diluted in HBSS to prepare different concentrations for each cell type: 20 µM JC10 for HL-60, 30 µM JC10 for U937 and 10 µM JC10 for HEK293T/17 cells. After each time point, cells were incubated with 300 µL of the corresponding JC10 solution for 15 min at 37°C. Afterward, cells were washed twice with HBSS and finally analyzed using FCM. The determination of cells with decreased mitochondrial membrane potential (ψ_m_) was realized as described previously.^24^

#### Nucleic acid staining

For cell staining of the nucleus, cells were cultured in eight-well Lab-Tek II chamber slides and seeded as follows: 1×10^5^ HL-60 cells in 0.5 mL RPMI 1640, 5×10^4^ U937 in 0.5 mL RPMI 1640 and 1.5×10^5^ HEK293T/17 in 0.5 mL DMEM. Cells were stained using 16.2 µM of the cell-permeable dye Hoechst. After an incubation time of 15 min at 37°C, cells were analyzed using Confocal Laser Scanning Microscopy (CLSM).

#### Lactate dehydrogenase (LDH) release

After incubating the cells with microcarriers in m:c ratios of 1:1, 5:1 and 10:1 for 24 h, the supernatant was collected to detect the release of the cytoplasmic enzyme LDH as an indicator for necrosis induction. Then, 1% Triton X-100 was used to induce maximal LDH release as a positive control. Cell supernatant was centrifuged at 500×*g* for 5 min to remove remaining cells. LDH assay was carried out in 96-well plates and each well contained the following substrates: 50 µL cell supernatant, 50 µL of 200 mM Tris buffer, 50 µL of 200 mM lithium lactate and 50 µL of a INT/PMS/NAD solution (with 4 vol% INT [33 µg/mL], 4 vol% PMS [9 µg/mL] and 92 vol% NAD [3.7 mg/mL]) Finally, the absorbance was measured.

### Experimental methods

#### Proton-induced X-ray emission (PIXE)

The PIXE measurements were carried out at the LIPSION facility by means of a 2.25 MeV proton beam.^30^ Object and aperture diaphragm diameters of 100 and 300 µm were used, respectively, to provide a beam current of 370 pA and a spot size of 1.3 µm. For all samples, a measurement time frame of 4 h was used to provide comparable information about Ca concentrations. The X-ray spectra were recorded with an HPGe detector from Canberra (95 mm^2^ active area). Two-dimensional elemental maps (50×50 µm) were recorded by scanning of the samples. The maps were analyzed with the GeoPIXE II software in order to extract the Ca-Kα line of the solid microparticle or microcapsule and the S-Kα line of the dextran sulfate multilayer constituent. The respective elemental maps were presented using a false color rendering (STD GAMMA-II) by setting the lowest pixel count per image as black color and the highest pixel count per image as white color. These values were adjusted for each individual image. Line profiles along a traverse, and Ca amount per solid microparticle or microcapsule were calculated and plotted separately.

#### Flow cytometry

FACS Calibur (Becton Dickinson, Franklin Lakes, NJ, USA) was used to detect and quantify fluorescence intensities of cells and microcarriers. For microcarrier/cell interaction studies, population of intact cells was selected in forward/sideward view and cells associated with RITC-labeled microcarriers were detected in FL2 channel (bandpass: 585±21 nm) when excited with a laser excitation wavelength of 488 nm. For viability measurements using JC10, all measured cells were considered. JC10 monomers were detected in FL1 channel (bandpass: 530±15 nm) after excitation at 488 nm and JC10 aggregates in FL2 channel. For each measurement, 10^4^ cells were detected in a region with specific forward scattering (FSC) and sideward scattering (SSC) values and further analyzed using Flowing Software.

#### Confocal laser scanning microscopy

For CLSM investigations, LSM 510 META (Zeiss, Oberkochen, Germany) was used. Nucleus (Hoechst33342) staining was detected using a laser excitation of 364 nm (krypton laser) with a bandpass filter of 385–470 nm. Detection of RITC-labeled microcarriers was carried out using laser excitation wavelength of 543 nm (helium/neon laser) and a bandpass filter of 560–615 nm.

#### Absorbance measurements

The microplate reader Tecan Infinite 200PRO (Tecan Group Ltd, Männerdorf, Switzerland) was used to monitor LDH absorbance at 503 nm. The kinetic LDH measurement was applied for 30 min with a time interval of 1 min. To calculate the relative LDH release, the positive control with maximum LDH release was set as 100%.

## Results and discussion

### Interaction kinetics

**Figure 1:**
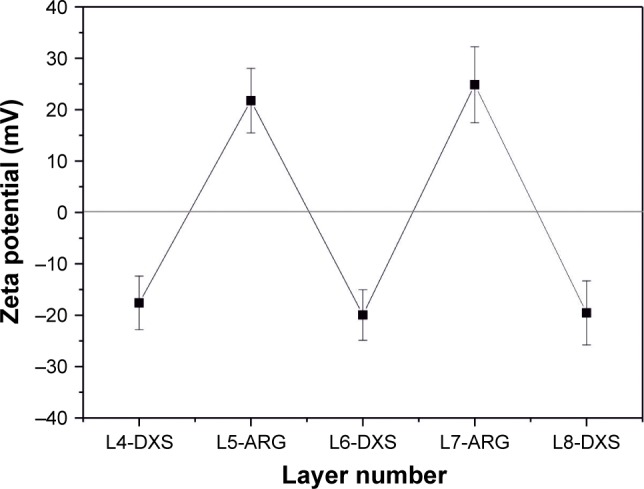
Zeta potential measurement of respective surface charges. **Notes:** Alternating potential profile of proceeding polymer assembly is shown after coating of outermost five layers with ARG and DXS. Polymer assembly ended either in position 9 (positively charged outermost ARG) or in position 8 (negatively charged outermost DXS). **Abbreviations:** ARG, poly-l-arginine hydrochloride; DXS, dextran sodium sulfate; L, layers.

LbL microparticles and capsules were prepared with at least six outermost layers of biopolymers ARG and DXS following coating with synthetic RITC-labeled PAH and PSS. Accuracy and stability of the preparation were investigated by zeta potential measurements of the respective outermost layer during assembly procedure. Figure 1 illustrates the alternating assembly of the oppositely charged biopolymers.

To investigate microcarrier/cell co-incubation, the interaction behavior was observed by FCM-based analysis in combination with confocal imaging (CLSM). Due to the fact that the FCM measurement, in general, allows no precise distinction between internalized microcarriers and microcarriers just adhering to the cell membrane, cells were treated in a specific manner: To remove adhering microcarriers in those experiments, all cell types were more intensively washed with enzyme (trypsin) compared to standard cell detaching procedure. In contrast, confocal images were used without any further treatment of the cells to cover all occurring microcarrier/cell interactions.

**Figure 2:**
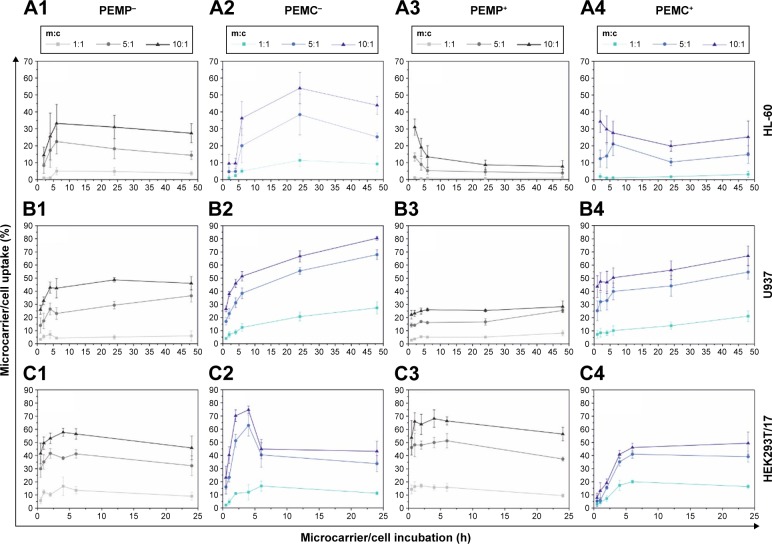
Quantitative FCM measurement of cellular uptake after application of LbL-microparticles (gray data lines) and LbL-microcapsules (blue data lines) with negative (columns 1 and 2) and positive (columns 3 and 4) surface charge in a time- and concentration-dependent manner. **Notes:** The upper panel (**A**) shows co-incubation with the neutrophil granulocyte-like cell line HL-60. Panel (**B**) represents the interaction of LbL-microcarriers with the macrophage-like cell line U937, whereas in the lower panel (**C**), co-incubation with the epithelial cells HEK293T is shown. All data points represent mean values with standard deviation, n≥6. **Abbreviations:** FCM, flow cytometry; LbL, layer-by-layer; m:c, microcarrier:cell; PEMC, polyelectrolyte-coated microcapsule; PEMP, polyelectrolyte-coated microparticle.

The results for quantitative analysis after FCM measurement are shown in Figure 2. Here, the microcarrier uptake is presented depending on the applied m:c ratio. Different co-incubation time intervals were chosen depending on specific interaction behavior of the different cell types. Stable interaction progress was reached after 48 h for the immune cells, while progress of epithelial cells was already completed by 24 h.

For direct comparison, different cell types as well as microcarriers with varying properties are shown in one image. Basically, the correlation of four main parameters has to be discussed: 1) professional and non-professional phagocytes, 2) LbL-microparticles (PEMPs) and LbL-microcapsules (PEMCs), 3) number of applied microcarriers, and 4) negative and positive surface charge of the applied LbL-microcarriers. Due to the fact that these parameters are strongly interconnected, a separate discussion of each parameter is not feasible.

However, some general similarities could be found. The uptake increases with the number of applied microcarriers and the interaction profiles are comparable for most combinations of investigated microcarrier/cell interactions: after an initial increase, a plateau is reached. Nevertheless, the total uptake rate as well as the time course for the initial increase varies considerably for all investigated cell types and LbL-microcarriers.

It could be observed in each graph in Figure 2 that an increasing m:c ratio results in an enhanced microcarrier/cell interaction and uptake, independent of the microcarrier’s properties.

The professional phagocyte cell line neutrophil-like HL-60 (Figure 2A) shows the best interaction rates with negatively charged microcarriers (PEMP^−^, PEMC^−^), in particular with negatively charged PEMCs which also provide the highest overall interaction rate with HL-60 cells (m:c 10:1, 54.03%±9.22%, 6 h). The typical course is followed here with an initial strong increase and transition into a plateau, although the time course is different. PEMPs reach the plateau much faster than PEMCs. Positively charged microcarriers (PEMP^+^, PEMC^+^) are less interactive and the profiles differ from the typical course. After an initial fast increase, the final plateau is lower compared to the interaction rates at the beginning of the time course. Here, we assume that a fast, massive microcarrier adhesion occurs that could not be completely neutralized by trypsin treatment and which will later pass into uptake or detachment.

The other professional phagocyte cell line, macrophage-representing U937 cells (Figure 2B), shows in parts a different interaction behavior compared to HL-60 cells. Again, negatively charged PEMPs and PEMCs show a higher interaction rate compared to their positively charged equivalents and the plateau is reached slower using PEMCs than using PEMPs. But in contrast to HL-60 cells, microcapsules generally dominate the interaction process independent of their surface charge, with the maximum interaction provided by the negatively charged PEMCs (m:c 10:1, 80.39%±1.48%, 48 h) and uptake of all microcarrier types progresses in the same way with an initial fast increase followed by a plateau.

The dominant uptake of PEMC is a result of the different stiffness of PEMPs and PEMCs in comparison with the cell’s function as professional phagocytes. PEMPs with a solid CaCO_3_ core exhibit a comparatively high Young’s modulus of 66 GPa,^31^ whereas PEMCs without a solid core have a Young’s modulus of only 0.24 GPa.^32^ This is in accordance with studies by other groups, which indicate that soft materials are internalized better and faster.^33^–^35^

Increased uptake of anionic particles of different sizes was described for professional phagocytes (such as macrophages) that was initiated probably by specific receptors, while other cells prefer cationic particles.^36^

The comparison of each microcarrier type interacting with different immune cells showed that macrophages present a higher uptake rate than neutrophil granulocytes. This could be explained by the different sizes of these cell types. Despite the fact that both types of immune cells represent professional phagocytes, it is more likely that macrophages with a size of about 20 µm are able to incorporate a large number of microcarriers than are HL-60 cells with a smaller size of only about 10 µm.

In contrast to the professional phagocytes, the epithelial cell line HEK293T/17 (Figure 2C) shows a more homogeneous outcome.

All applied m:c ratios result in a plateau after 6 h of co-incubation as well as in a comparable interaction rate independent of the LbL-microcarrier modification, PEMPs or PEMCs, negative or positive charge (after 24 h of co-incubation in the case of m:c 10:1 ratio: PEMP^−^ 45.80%±9.04%; PEMP^+^ 56.42%±5.11%; PEMC^−^ 43.11%±7.68%; PEMC^+^ 49.40%±8.54%). Again, PEMCs (negatively charged, high m:c ratio) cause an abnormality in interaction kinetics. During the first 6 h, a strong increase can be detected; the subsequent plateau is considerably lower (as already shown for PEMC^+^, HL-60, high m:c ratio), which may again be caused by a massive microcarrier adsorption and incomplete removal by trypsin.

**Figure 3:**
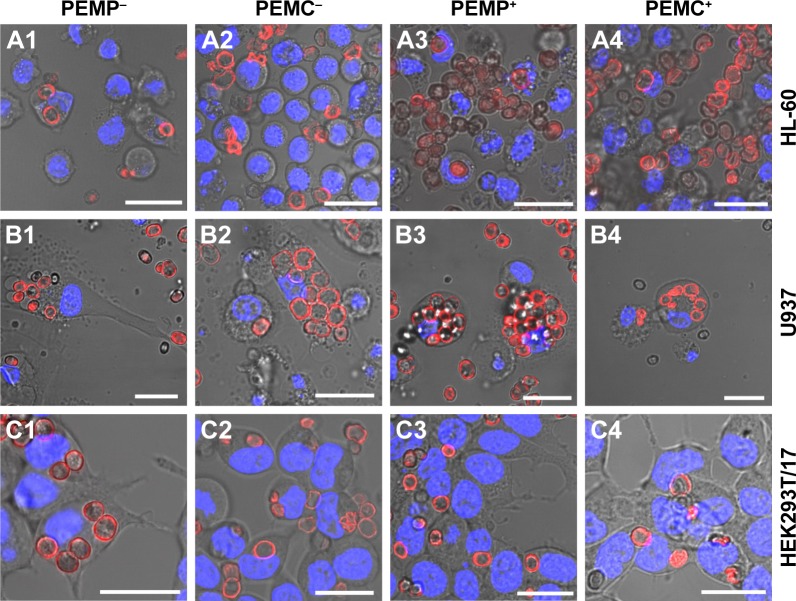
Representative CLSM images after co-incubating LbL-microparticles (columns 1 and 3) and LbL-microcapsules (columns 2 and 4) with negative (columns 1 and 2) or positive (columns 3 and 4) surface charge and different cell types (**A**: HL-60 cells, **B**: U937 cells and **C**: HEK293T cells) to differentiate internalized LbL-microcarriers and LbL-microcarriers just adhering to the cell surface. **Note:** Scale bars: 20 µm. **Abbreviations:** CLSM, confocal laser scanning microscopy; LbL, layer-by-layer; PEMCs, polyelectrolyte-coated microcapsules; PEMPs, polyelectrolyte-coated microparticles.

Further analysis of the microcarrier/cell interaction after 24 h of co-incubation was carried out using CLSM (Figure 3) to obtain more insight into the comprehensive interaction process. These qualitative investigations reveal more outcomes concerning: 1) the effective internalization of LbL-microcarriers in correlation with the quantitative FCM analysis (Figure 2); and 2) an insight into the amount and distribution of internalized LbL-microcarriers depending on the cell type. In our experiments, transmitted light images were used to show the observed cell layer section, whereas the fluorescence images enable visualization of labeled LbL-microcarriers in the same focal plane as the stained cell nuclei.

Representative results in Figure 3 support our findings from FCM investigations. Professional phagocytes show a high incorporation behavior dominated by macrophages (U937), whereas the cell size differences and subsequent number of incorporated microcarriers can be clearly distinguished. Concerning HL-60 (Figure 3A), negatively charged microcarriers (PEMP^−^, PEMC^−^) show an increased uptake effect compared to positively charged microcarriers, as most of the co-incubated microcarriers are internalized. Despite a higher local density of positively charged microcarriers as displayed for PEMP^+^ and PEMC^+^, most of them are located in the extracellular space or still attached to the membrane as the different focal plane illustrates. Regarding U937 (Figure 3B), a general high uptake (internalized microcarriers ascertained by number) can be observed, which renders it difficult to estimate differences in uptake. Uptake of HEK293T/17 cells illustrates a more uniform behavior independent of the microcarrier charge and nature (Figure 3C) as comparable local cell and carrier number suggests.

### Impact on cell viability

Besides the studies of internalization kinetics, another important point is the investigation of the impairments on cell viability. In such investigations, all components of the respective drug delivery system have to be considered. For our system, impairments of cell viability can arise mainly from the CaCO_3_ core and/or the multilayer material which can change its properties over time and during the cellular internalization process.^10^ Degradation of the LbL-multilayer under cellular conditions can occur as a consequence of changes in pH or salt concentration or due to an enzymatic degradation, considering the fact that the multilayer consists of biopolymers such as peptides and polysaccharides.

But while PEMPs and PEMCs are equipped with the same multilayer material and thickness to allow a direct comparison in the following experiments, the core material status varies from solid CaCO_3_ template to hollow capsules, which needs to be discussed in more detail beforehand. On one hand, an intracellular decomposition of the solid CaCO_3_ core is unlikely due to its low solubility product and poor water solubility. Nevertheless, since Ca^2+^ is a key element of many cellular signaling cascades, this could be a critical aspect after capsule preparation. So, on the other hand, incompletely removed CaCO_3_ or Ca^2+^, such as that remaining within the core or intercalating into the multilayer during penetration, may drastically affect cell viability.^28^

**Figure 4:**
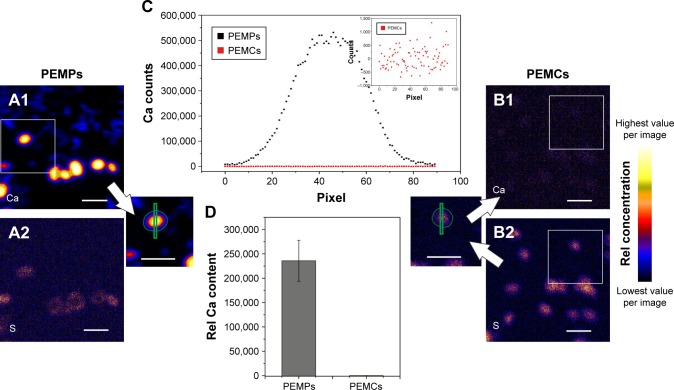
PIXE analysis of PEMPs and PEMCs to detect free Ca after CaCO_3_ core dissolution. **Notes:** The Ca-maps are shown in **A1** (PEMPs) and **B1** (PEMCs), whereas the S-maps are shown in **A2** (PEMPs) and **B2** (PEMCs). PEMP Ca profile lines and Ca outlines (amount) were taken directly from the Ca map, whereas PEMCs were located using S-map, and profile lines and outlines were translated into the Ca map. Representative Ca-profiles (**C**) of PEMPs (black) and PEMCs (red) indicate the complete CaCO_3_ removal during core dissolution, which is verified by the relative Ca content (**D**). Scale bars: 10 µm. **Abbreviations:** CaCO_3_, calcium carbonate; PEMC, polyelectrolyte-coated microcapsule; PEMP, polyelectrolyte-coated microparticle; PIXE, proton-induced X-ray emission; Rel, relative.

Therefore, we investigated the removal status of CaCO_3_ after standard core dissolution procedure by proton-induced X-ray emission (PIXE) to allow an estimation of the influence of Ca^2+^ in our further experiments. This two-dimensional method facilitates the detection of trace elements with exceptional high sensitivity and was applied to locate potentially remaining Ca.^30^ As already shown, mapping of microcarriers can be used to evaluate elemental composition and spatial elemental distribution, so presence or lack of template material or remaining components could be easily detected.^37^ Results are shown in Figure 4. In Figure 4A and B, elemental maps of calcium (Ca-map, A1, B1) and sulfur (S-map, A2, B2) are presented. The S-maps serve as an equivalent for transmission images, since S is an unchanging microcarrier constituent due to the dextran sulfate component within the multilayer. Thus, even with completely removed CaCO_3_, the microcapsules and their location remain visible and line profiles along a traverse as well as Ca content within the multilayer wall can be estimated.

Images A1 and B1 clearly illustrate the fate of CaCO_3_ after core dissolution. Following a representative PEMP and PEMC, as shown in Figure 4C, the Ca profile of a solid microparticle is reduced to background intensity in the case of the PEMC. The inset shows the Ca line profile of the micro-capsule in more detail. Mean Ca content of all investigated PEMPs and PEMCs is then plotted in Figure 4D, presenting the total loss of Ca during the CaCO_3_ dissolution process using ARG and DXS as multilayer constituents.

Thus, an underlying negative effect of free Ca^2+^ (by intercalating into the LbL-multilayer) can be ruled out in the case of PEMCs, which allows a direct comparison of the LbL-multilayer and the used CaCO_3_ core in combination with the multilayer to determine the impact of the DDS components subsequently.

**Figure 5:**
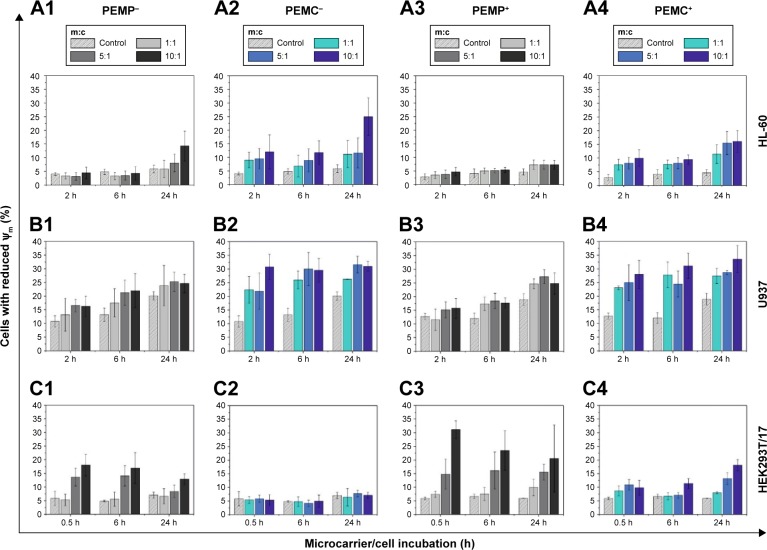
Detection of early apoptotic signaling by FCM measurements after co-incubating different cell types (**A**: HL-60 cells, **B**: U937 cells and **C**: HEK293T cells) with LbL-microparticles (gray bars) and LbL-microcapsules (blue bars) with negative (columns 1 and 2) or positive (columns 3 and 4) surface charge in a time- and concentration-dependent way. **Notes:** Cells with reduced mitochondrial membrane potential (Ψ_m_) are referred to as apoptotic cells. All data points represent mean values with standard deviation, n≥6. **Abbreviations:** FCM, flow cytometry; LbL, layer-by-layer; m:c, microcarrier:cell; PEMCs, polyelectrolyte-coated microcapsules; PEMPs, polyelectrolyte-coated microparticles.

**Figure 6:**
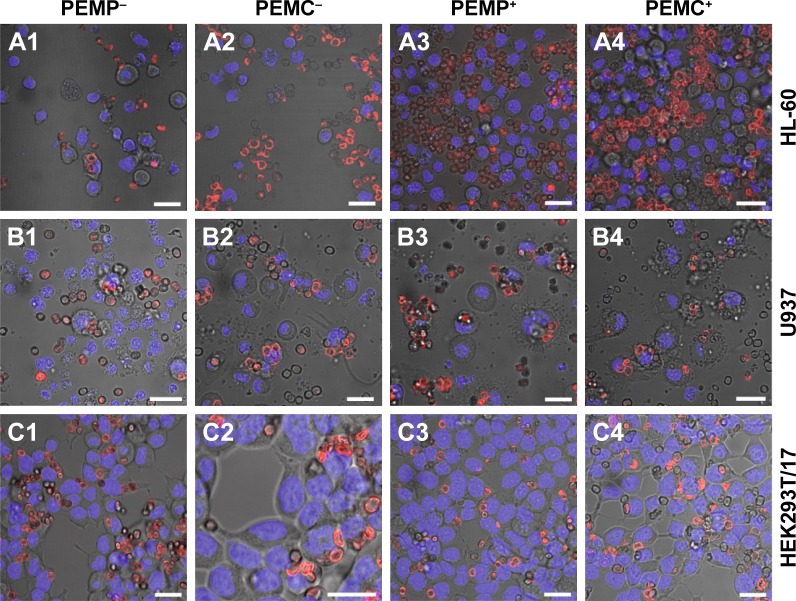
Detection of late apoptotic signaling by Hoechst staining analyzed with CLSM after a co-incubation time of 24 h for different cell types (**A**: HL-60 cells, **B**: U937 cells and **C**: HEK293T cells) with LbL-microparticles (columns 1 and 3) and LbL-microcapsules (columns 2 and 4) with negative (columns 1 and 2) and positive (columns 3 and 4) surface charge. **Note:** Scale bars: 20 µm. **Abbreviations:** CLSM, confocal laser scanning microscopy; LbL, layer-by-layer; PEMCs, polyelectrolyte-coated microcapsules; PEMPs, polyelectrolyte-coated microparticles.

**Figure 7:**
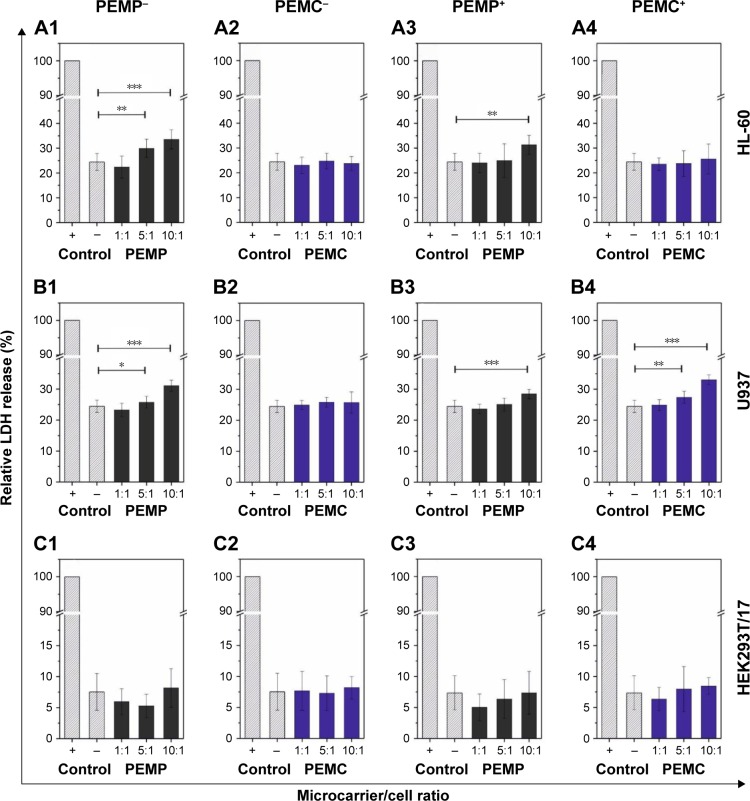
Analysis of necrotic signaling by detecting the release of the cytoplasmic enzyme LDH after a co-incubation time of 24 h. **Notes:** Different cell types (**A**: HL-60, **B**: U937 and **C**: HEK293T cells) were co-incubated in a concentration-dependent way with LbL-microparticles (gray bars) and LbL-microcapsules (blue bars) with a negative (columns 1 and 2) or a positive (columns 3 and 4) surface charge. A positive control of each cell line, cells without microcarrier co-incubation and treated with Triton™ X-100 for cell membrane disruption, was set as 100% LDH release. All data points represent mean values with standard deviation, n≥8. Statistical analysis was carried out using a two-tailed student’s *t*-test: *p≤0.05, **p≤0.01, ***p≤0.001. **Abbreviations:** LbL, layer-by-layer; LDH, lactate dehydrogenase; PEMCs, polyelectrolyte-coated microcapsules; PEMPs, polyelectrolyte-coated microparticles.

To investigate the impact on cell viability, in this study, different viability approaches were applied to cover several intracellular signaling pathways: early and late apoptotic signaling as well as necrotic signaling. The first step aims to explore the detection of early apoptosis, which can be investigated by monitoring changes of the mitochondrial membrane potential. This early alteration during an apoptotic process could be investigated by staining the cells with JC10 and analyzing with FCM (Figure 5). In the next step, late apoptotic signaling was investigated using Hoechst staining to visualize nucleus alterations by CLSM (Figure 6). Finally, the induction of necrosis was monitored using an LDH assay and detected by photometry (Figure 7). Again, the investigations were carried out in a time- and concentration-dependent way with different cell types (neutrophil granulocytes, macrophages and epithelial cells) and LbL-microcarriers with varying properties.

Investigating the impact on early apoptotic signaling (Figure 5), the obtained results show the amount of cells with reduced mitochondrial membrane potential (Δψ_m_, compared to positive control), which correlates with the amount of apoptotic cells. The investigated time points were chosen according to the microcarrier’s intracellular processing: the first time point represents the state of the beginning of the microcarrier/cell interaction, whereas the second time point defines a state with almost completed internalization. The maximum incubation time of 24 h was chosen according to the limited lifetime of the investigated immune cells.

For the differentiated HL-60 cells, which represent neutrophil granulocytes, incubation with negatively charged PEMPs (Figure 5A1) induces after 24 h, and only in the case of 10:1, a slight negative impact on cell viability (control 5.87%±1.41%; m:c 10:1, 14.25%±5.48%). In contrast, PEMCs with a negative surface charge (Figure 5A2) seem to induce more impairments (up to 24.95%±6.93% apoptotic cells), as can be observed at each incubation time point and each applied m:c ratio.

Similar results were observed after co-incubation of HL-60 cells with positively charged LbL-microcarriers (Figure 5A3 and A4). But other than that, the positively charged LbL-microcarriers induced less impairments on the viability of HL-60 cells, compared to LbL-microcarriers with a negative surface charge. This unexpected outcome could be explained by the considerably higher interaction rate of LbL-microcarriers with negative surface charge (Figure 2): a higher amount of internalized LbL-microcarriers is expected to induce higher impairment of cell viability.

However, both positively and negatively charged microcapsules always induce a higher viability reduction than solid microparticles. While ruling out remaining Ca (Ca^2+^) as a potential source of toxicity (Figure 4), the effect could then be based on differences in uptake behavior (Figure 2A1 and A2).

Results for the second type of immune cells, macrophages-representing U937 cells, are in parts similar. Again, co-incubation with PEMCs (both negatively and positively charged) results in a higher amount of apoptotic cells compared to the incubation of U937 cells with PEMPs. Even so, for every incubation time point, a significant impact on cell viability could be observed, independent of the applied m:c ratio and PEMP or PEMC application (Figure 5B2 and B4). These different results for HL-60 and U937 cells could be a result of the varying interaction kinetics: U937 cells show an interaction with LbL-microcarriers already beginning at an earlier time point (Figure 2). Additionally, U937 cells show in total a much higher interaction with LbL-microcarriers, which again correlates with more impairments in cell viability compared to HL-60 cells.

For immune cells (HL-60 and U937), one of the natural features is to phagocytose and eliminate foreign objects, so the question arises as to what happens to the epithelial cells (HEK293T/17) after incorporating LbL-microcarriers. As shown in Figure 5C, the results are now completely different compared to those of the immune cells. High PEMP/cell ratios induce a comparatively severe impact on cell viability in the case of PEMPs with negative surface charge (Figure 5C1), which is even more prominent in the case of PEMP^+^ (Figure 5C3). These results are in accordance with other studies investigating charge-dependent cell interactions.^38^,^39^ Due to the lower cell interaction after co-incubation with PEMCs, the amount of apoptotic cells is also lower compared to HEK293T/17 cells incubated with PEMPs.

In the next step, investigations focus on the detection of late apoptotic signaling after co-incubating the different cell types with varying LbL-microcarriers, which is shown by representative CLSM images in Figure 6. Based on the fact that late apoptosis detected by nucleus alterations occurs in a delayed manner after internalization and processing of the LbL-microcarriers, the following investigations regarding the microcarrier impact on cell viability were carried out after the maximum incubation time of 24 h.

Compared to the control (cells without microcarrier co-incubation), both PEMPs and PEMCs caused no late apoptotic signaling in the investigated cell types. Individual fragmented cell nuclei could be equally observed in the control cells, and are therefore not caused by co-incubation with LbL-microcarriers.

Finally, to detect necrotic signaling, the release of the cytoplasmic enzyme LDH was measured, which is a conventional characteristic for cell necrosis and is used in this study to quantify the microcarrier impact. Again, the different cell types were co-incubated with LbL-microcarriers for an incubation time of 24 h to uncover the maximum effect (Figure 7).

The incubation of differentiated HL-60 cells with high amounts of PEMPs, both negatively and positively charged, leads to a slight increase in LDH-release, whereas the incubation with PEMCs exhibits no differences compared to the control (cells without microcarrier co-incubation). These results are contrary to the investigations regarding early apoptotic signaling, but not contradictory, due to the different, and not in general, connected signaling pathways. Regarding differentiated U937 cells, which represent macrophages, a slight negative impact on necrotic signaling could be observed only in the case of high PEMP/cell ratios (both, negative and positive charges) as well as high PEMC/cell ratios (positive charge) (Figure 7B). Compared to immune cells, co-incubation of HEK293T/17 cells with different LbL-microcarriers induces no obvious impairment in cell necrosis (Figure 7C).

Based on the aforementioned results, Table 1 shows a compilation of the extracted conditions for an application of such LbL-microcarriers as a drug delivery system in different cells, considering the best results regarding uptake rates and impairments in cell viability. However, different fields of application may set their emphasis on specific significant parameters, which changes the focus of the selection. As can be seen, different applications require different microcarrier equipment and maximum co-incubation time. Similar uptake rates by different cells under convenient cellular conditions can be reached by selection of stiffness, charge and cell–carrier ratio.

## Conclusion

Our comprehensive DDS investigations concentrate only on a narrow class of prominent parameters of DDS design, materials and properties. With the investigations conducted under comparable conditions, the results impressively illustrate that even within such a narrowly defined class, the physicochemical properties of the investigated LbL-microcarriers as well as the cellular properties have a huge impact on the uptake behavior, interaction rate and viability.

The variation of surface charge and stiffness (solid microcarriers and hollow microcapsules) results in uptake rates which differ up to 50 percentage points (granulocytes, macrophages) and in various uptake kinetics and profiles, reaching the maximum point of interaction at different time points. Also the effects of positive or negative surface charge or application number vary from one cell type to another.

Even equally equipped microcarriers can induce completely different uptake behavior and kinetics; thus, carrier–cell interaction behavior cannot be translated from one cell type to another. Also, cell viability can be differently affected by comparable DDS parameters.

This emphasizes the necessity of a strong selection of suitable parameters to fulfill specific demands and to clearly apply to the needs of the respective studies: many proof-of-principle investigations, highlighting specific structural or functional aspects of nano- or microcarriers with respect to transport, release and effect of active agents into cells, are conducted by using randomly chosen cell types and basic DDS equipment.

While we have summarized only a few important aspects of LbL-microcarriers and cell types, the drastic differences in microcarrier/cell behavior suggest it is important to establish appropriate conditions already of the respective basis DDS even before the DDS is finally equipped with an active agent.

## Figures and Tables

**Table 1:** Optimal microcarrier parameters for cell application considering microcarrier properties, uptake rates as well as cell viability in response to microcarrier co-incubation

	Optimal parameters				
	m:c ratio	Stiffness/carrier type	Charge	Co-incubation time	Uptake
HL60	5:1	Capsule	Negative	24 h	40%
U937	10:1	Particle	Negative	24 h	45%
HEK293T/17	10:1	Capsule	Negative	6 h/24 h	45%
	5:1	Capsule	Positive	6 h	40%

**Abbreviation:** m:c, microcarrier:cell.
